# Adverse Ocular Effects of Systemic Medications

**DOI:** 10.3390/life13030660

**Published:** 2023-02-28

**Authors:** Michael B. Green, Jay S. Duker

**Affiliations:** 1 Department of Ophthalmology, Boston University School of Medicine, Boston, MA 02118, USA; 2 Department of Ophthalmology, New England Eye Center, Tufts Medical Center, Boston, MA 02111, USA

**Keywords:** ophthalmology, medication adverse effects, screening

## Abstract

While ocular complications of systemic medications are uncommon, it is important to recognize that vision-threatening toxicities can occur. This review details the vision-threatening adverse effects of a select group of commonly prescribed systemic medications and describes the recommended screening guidelines for those that are particularly high risk.

## 1. Introduction

Ocular complaints often represent a significant diagnostic challenge to non-ophthalmic healthcare providers, making it difficult to parse out serious vision-threatening conditions from those more benign in nature. While a comprehensive eye examination cannot be performed without proper training, obtaining a thorough history is useful in forming a differential diagnosis as well as triaging a referral to an eye care specialist. In particular, considering a patient’s medication regimen may raise suspicion of ocular side effects related to systemic medications. In this article, we review vision-threatening complications of commonly prescribed systemic medications and discuss the screening guidelines for certain high-risk medications. This review is among the most comprehensive peer-reviewed overviews of the ocular side effects of systemic medications and adds to the limited literature on the topic.

## 2. Corticosteroids

Corticosteroid medications (e.g., prednisone and dexamethasone) are known to put patients at increased risk for premature cataract formation (opacification of the crystalline lens) as well as increased intraocular pressure, potentially leading to glaucoma (optic nerve damage related to high intraocular pressure that typically causes progressive loss of peripheral vision). Corticosteroids are classically known to cause the development of cataracts in the posterior aspect of the lens (posterior subcapsular cataracts), which tend to be more visually significant than other forms of cataracts. Fortunately, surgical treatment of these cataracts is similar to those of other forms. While topical and periocular corticosteroids confer the highest risk of cataract formation, systemic corticosteroids can have a similar effect, particularly when taken at moderate to high doses over many years [1]. The association of cataracts with inhaled and topical dermatologic corticosteroid use is more controversial [2,3,4].

More concerning, however, is the risk of glaucoma associated with corticosteroid use, which, unlike cataracts, causes irreversible vision loss. Furthermore, while cataracts tend to cause perceptible visual symptoms early on, the gradual vision loss that occurs from glaucoma typically goes unnoticed by patients until it has already reached advanced stages. It has been estimated that intraocular pressure (IOP) steroid response occurs in approximately 1/3 of the population [5]. The extent of IOP elevation is dependent on the method of administration and steroid potency, with topical corticosteroid eyedrops and periocular corticosteroid injection being the highest risk [6]. Similar to corticosteroid-induced cataracts, steroid-induced glaucoma typically only occurs in cases of chronic steroid use at moderate to high doses [6]. Additionally, as with cataracts, the association between inhaled and topical dermatological corticosteroid treatment with intraocular pressure elevation is controversial [2,3,4]. Given the asymptomatic nature of steroid-induced ocular hypertension, referral to an eye care provider for IOP monitoring is recommended for those using steroids chronically.

Finally, a rarer association with corticosteroids is the occurrence of central serous chorioretinopathy (CSCR), which typically affects young healthy males and results in a central area of blur in one eye.

## 3. Chloroquine and Hydroxychloroquine

Chloroquine and hydroxychloroquine, which are used for their anti-malarial and immunosuppressive properties, have the potential to cause irreversible retinal damage with prolonged use. By binding to melanin in the retinal pigment epithelium (RPE), these medications can accumulate underneath the retina and cause permanent toxicity to the overlying macula (which is the region of the retina responsible for central vision). In non-Asian patients, the center of the macula is typically affected, whereas Asian patients can develop para-central damage initially. This toxicity classically causes a “bull’s eye maculopathy” on fundus examination, though vision loss occurs well before the development of this examination finding. The risk of toxicity is lower for patients taking <5.0 mg/kg/day of hydroxychloroquine or <2.3 mg/kg/day of chloroquine, though toxicity can still occur with long-term use even at these doses [7]. The risk of maculopathy at recommended doses is <1% after 5 years and <2% after 10 years but then rises significantly to 20% after 20 years of use [8]. Additionally, concomitant renal or hepatic disease and the concurrent use of tamoxifen elevate the chances for the development of toxicity.

Routine asymptomatic screening is critical in these patients as toxicity is irreversible once it occurs and may progress even after cessation of treatment. For patients without risk factors for toxicity, the American Academy of Ophthalmology (AAO) [9] recommends baseline screening prior to or within one year of initiation to rule out preexisting maculopathy (which may mask the onset of toxicity) followed by annual screening examinations beginning after 5 years of treatment. Patients at higher risk of toxicity should be screened earlier and more frequently based on clinical judgment. Recommended screening includes visual acuity testing, fundus examination, optical coherence tomography (OCT), and visual field testing (10-2 testing for non-Asian patients and 24-2 or 30-2 for Asian patients). Multifocal electroretinography and fundus autofluorescence imaging can also be used. These aforementioned medications should be stopped at the first sign of retinal toxicity.

## 4. Pentosan Polysulfate Sodium

Pentosan polysulfate sodium (PPS) is currently the only oral treatment for interstitial cystisis/bladder pain syndrome approved by the US Food and Drug Administration (FDA). In 2018, a case series of 6 patients initially described the development of macular disease after long-term use of PPS [10]. PPS maculopathy has been shown to occur after a median of 14.5 years (range 3–21.9 years) of treatment [11] and is believed to be dose-dependent with rates of 12.7%, 30.0%, and 41.7% in patients with a cumulative exposure of 500–999 g, 1000–1500 g, and greater than 1500 g, respectively [12]. Patients most often present with difficulty reading and delayed dark adaptation [10]. While visual acuity may remain preserved early on, late-stage toxicity can result in significant vision loss secondary to macular edema, RPE atrophy, or choroidal neovascularization [11]. It is not unusual for such late-stage patients to carry the diagnosis of dry age-related macular degeneration. The AAO has not yet released screening guidelines for these patients, though other groups have suggested obtaining a baseline examination with fundus autofluorescence, near-infrared, and OCT imaging prior to initiation of treatment followed by annual monitoring as the cumulative dose approaches 500 g (approximately 5 years with standard dosing) [13]. Providers should consider avoiding this medication in patients with baseline macular disease which may increase the risk of toxicity or mask its onset. The medication should be stopped at the first sign of retinal toxicity.

## 5. Amiodarone

While the most common ocular side effect of the antiarrhythmic medication amiodarone are corneal deposits, these lesions are normally not visually significant. More concerning is the rarer optic neuropathy that can result from amiodarone use. Affecting an estimated 1.76% of amiodarone users [14], amiodarone-associated optic neuropathy (AAON) causes insidious, slowly progressive vision loss that usually becomes symptomatic within one year of amiodarone initiation (median 6 months; range 1–84 months) [15]. Patients typically have bilateral involvement, though unilateral cases do occur (approximately 20% of cases) [14]. Additionally, patients with bilateral disease may present with unilateral symptoms or may be completely asymptomatic initially [15]. Discontinuation of amiodarone should be considered at the first sign of optic neuropathy. After discontinuation, approximately 60% have an improvement in vision, whereas 20% remain the same and 20% continue to lose vision [15]. Disc edema may persist for a few months after discontinuation given the 100-day half-life of amiodarone. There is currently no formal guideline for screening AAON, but some have recommended multiple examinations within the first year and then annually thereafter [15].

## 6. Ethambutol

Ethambutol is an antibiotic used in the treatment of mycobacterial infections including *Mycobacterium tuberculosis* (TB). Ethambutol is well-known to cause dose-dependent bilateral optic neuropathy with a prevalence of 1% at doses of ≤15 mg/kg/day, 5–6% at 25 mg/kg/day, and 50% at doses of 60–100 mg/kg/day [16]. This adverse effect is thought to occur due to the disruption of mitochondrial oxidative phosphorylation [17]. Ethambutol optic neuropathy (EON) typically causes insidious bilateral, painless, and symmetric loss of central vision with associated dyschromatopsia and often presents between 2 and 8 months after initiation of therapy, though it can occur at any time [16]. Because EON causes retrobulbar optic neuropathy, optic nerve appearance will be normal in the acute phase, with late-onset nerve pallor occurring if the disease is allowed to progress. Patients of older age and those with hypertension or renal disease are at increased risk, making it critical that clinicians use renal dosing when indicated and adjust for any changes in weight during the course of treatment [18]. While the AAO does not provide formal guidelines on the screening of EON, a recent consensus statement from India recommends baseline visual acuity, color vision, contrast sensitivity, visual field, and retinal nerve fiber layer OCT testing followed by repeat assessments every 2–3 months [19]. Patients should also be encouraged to regularly perform visual acuity and Amsler grid testing at home and notify their provider of any changes. The only treatment for EON is prompt discontinuation of ethambutol, with 30–64% of patients showing some visual improvement when detected early [17]. Maximal recovery may take up to 12 months after treatment cessation [20], though patients who were initially treated for greater than 6 months are more likely to suffer irreversible damage [21].

## 7. Topiramate

Topiramate is a sulfamate-substituted monosaccharide indicated for the treatment of seizure disorders and as migraines prophylaxis but with a myriad of off-label uses including weight loss, alcohol use disorder, binge eating disorder, bulimia nervosa, idiopathic intracranial hypertension (IIH), and essential tremor. Although the mechanism remains unclear, topiramate is known to cause transient myopia (nearsightedness) and acute angle-closure glaucoma. It is believed that topiramate causes increased permeability of the choroidal vasculature of the eye which leads to choroidal effusion and ciliary body swelling [22]. This causes increased lens thickness and anterior displacement of the lens, which changes the eye’s refractive error. These changes also cause anterior displacement of the lens-iris diaphragm and subsequent shallowing of the anterior chamber, which causes obstruction of the drainage pathway of the eye (i.e., closure of the angle) leading to a sudden increase in IOP. If not promptly treated, this high IOP can cause permanent glaucomatous damage to the optic nerve. Topiramate-induced acute angle closure (TIAAC) has an estimated prevalence of 3 per 100,000 patients [23]. Classic symptoms of angle closure include significant pain, headache, nausea and vomiting, decreased vision, and eye redness. A review of 86 cases of suspected TIAAC found that 96.5% of cases were bilateral and simultaneous, and 85% occurred within the first 2 weeks (range 1 to 49 days) of topiramate initiation (with 5 cases occurring within hours of doubling their prior topiramate dose) [24]. The same study also reported cases of TIAAC at a wide range of dosing, suggesting the adverse effect may not be dose-dependent. Treatment of this condition includes urgent IOP-lowering medications—although miotics (i.e., pilocarpine) should be avoided as this may exacerbate the condition by causing a pupillary block. Laser iridotomy or iridectomies are not indicated given this condition does not cause a pupillary block. While asymptomatic screening is not useful for this condition, vision changes in patients on topiramate should prompt urgent referral to an ophthalmologist.

## 8. Tetracyclines

Tetracyclines are a class of antibiotics with both antimicrobial and anti-inflammatory effects used acutely for bacterial infections and chronically for dermatologic conditions such as acne vulgaris and rosacea. Agents in this class include minocycline, tetracycline, and doxycycline. The use of these medications has been associated with intracranial hypertension (IH)—also known as pseudotumor cerebri. Typical symptoms of IH include headache, nausea and vomiting, pulsatile tinnitus, transient visual obscurations, decreased vision, and diplopia. In most cases, this elevated intracranial pressure (ICP) is associated with bilateral papilledema. If this papilledema becomes severe enough, it can lead to permanent vision loss. Although the mechanism of tetracycline-associated IH (T-IH) is not fully understood, decreased cerebrospinal fluid absorption at the arachnoid villi has been suggested [25]. A review of 96 cases of T-IH found that minocycline was the most commonly implicated medication (64.6%), followed by tetracycline (21.9%) and doxycycline (13.5%), and that the mean length of therapy prior to the onset of IH symptoms was 18.9 weeks (range of several days to 4 years) [26]. There is still some debate as to whether or not tetracyclines cause IH primarily or if they are just an aggravating factor in patients already predisposed to developing the condition. Young age, female sex, and weight gain are all strong risk factors for the development of idiopathic IH (IIH), and while many studies have found similar age and female predilections (~90% female) among cases of T-IH and IIH, the association between T-IH and obesity is less consistent [26]. Additionally, the incidence of IH in tetracycline users has been found to be significantly higher than in the general population (63.9 vs. <1 per 100,000 person-years) [27]. Finally, T-IH patients have been shown to be 4 times less likely to recur after tetracycline cessation and acetazolamide treatment, which suggests the tetracyclines may be the primary cause of increased ICP in many cases [26]. There is no role for asymptomatic screening in this condition, though clinicians should consider patients’ risk factors for IIH prior to treatment initiation and obtain urgent ophthalmology or neurology referral if symptoms of IH develop.

## 9. Vitamin A and Retinoids

Vitamin A and its retinoid derivatives—which are used primarily in the treatment of inflammatory dermatologic conditions such as severe or refractory acne vulgaris as well as acute promyelocytic leukemia—can also cause increased ICP. IH is known to occur as a result of hypervitaminosis A from excessive supplementation [28] and shows a dose-dependent and cumulative effect [29]. A review of 179 cases of isotretinoin-associated IH (I-IH) [30] found a median time of 2.3 months from initiation of isotretinoin therapy to symptoms of I-IH. Resolution of I-IH occurred over a matter of weeks to months after discontinuation. Screening for retinoid-associated IH is not routinely recommended, though some have suggested periodic ophthalmologic examination in patients taking retinoids for greater than 6 months [31]. Additionally, simultaneous use of retinoids with vitamin A supplementation or tetracyclines should be avoided and caution should be exercised in patients with risk factors for IIH (young, obese females).

## 10. Niacin

Niacin (nicotinic acid, vitamin B_3_), which is used to help reduce cholesterol and triglyceride levels, can cause maculopathy in a minority of patients when taken in high doses. This adverse effect has been reported in patients taking as little as 1.5 g daily [32,33], which is well below the maximum dosing of 6 g daily for the treatment of dyslipidemia. At these doses, niacin can cause edema to develop within the retinal layers of the macula. Patients with macular edema typically experience blurred or dulled central vision but can also report visual distortions (metamorphopsia). Patients who develop niacin maculopathy typically do so within weeks to months of starting treatment and usually suffer mild to moderate vision loss [33]. Fortunately, this adverse effect is rare, with an estimated incidence of 0.67%, and resolves within 4 to 8 weeks after niacin discontinuation [33]. While visual function often returns to normal with a resolution of macular edema, chronically untreated macular edema can lead to irreversible vision loss. While routine screening is unnecessary, prompt ophthalmologic evaluation should be performed in patients taking niacin who experience vision changes.

## 11. Fingolimod

Fingolimod is an immunomodulatory medication used in the treatment of relapsing-remitting multiple sclerosis and has been shown to cause macular edema in some patients. Studies report an incidence of ~0.4% at the typical 0.5 mg/day dosing, with rates increasing at higher doses or in those with preexisting uveitis or diabetes mellitus [34]. Sixty-eight percent of patients who develop fingolimod-associated macular edema (FAME) do so within 3–4 months of treatment initiation, and 84% of cases resolve with fingolimod discontinuation [35]. As with other causes of macular edema, patients will usually present with decreased central vision, though some may be asymptomatic. Patients being treated with fingolimod, particularly those with a history of diabetes or uveitis, should obtain a baseline eye examination prior to initiation of treatment followed by a repeat examination after 3–4 months of therapy [36]. Patients can be monitored annually thereafter. If possible, therapy should be discontinued if macular edema develops. In cases where fingolimod must be continued, FAME has been shown to occasionally respond with various corticosteroid and non-steroidal treatments [34].

## 12. Vigabatrin

Vigabatrin is an antiepileptic used in the treatment of seizure disorders in childhood and adulthood. It has the potential to cause irreversible dose-dependent [37] visual field loss, which led to its FDA approval being accompanied by a mandatory Risk Evaluation and Mitigation Strategy program. While not fully understood, vigabatrin-associated visual field loss (VAVFL) is thought to occur as a result of toxicity to retinal photoreceptor and ganglion cells [38]. Some level of field loss is estimated to occur in 30–50% of patients taking vigabatrin [39], with most cases occurring in those on treatment for over one year [40]. Vision loss from vigabatrin typically begins as peripheral field constriction and is therefore usually asymptomatic at the onset. As a result, routine screening is required in those patients, which involves baseline fundus examination with visual field, electroretinography, or retinal nerve fiber layer OCT testing prior to initiation followed by testing every 3 months during therapy and then 3 to 6 months after discontinuation [40,41]. Unfortunately, these tests may be difficult to perform or interpret in young children. Withdrawal of therapy should be strongly considered in cases where visual field loss is suspected.

In addition to the medications detailed above, there are many other ocular complications associated with systemic medications many of which are summarized in Table 1. Screening guidelines for the detection of ocular toxicity in a selection of high-risk medications are outlined in Table 2. Incidences of vision-threatening adverse effects of select chemotherapeutic agents are reviewed in Table 3.

## 13. Conclusions

This study provides an up-to-date and wide-ranging overview of the ocular side effects of systemic medications. In general, ocular complications of systemic medications are uncommon. However, it is important to recognize medications that may place patients at higher risk of potentially vision-threatening complications and to identify which medications require routine screening by an ophthalmologist for evaluation of ocular toxicity.

## Figures and Tables

**Table 1:** Adverse Ocular Effects of Select Systemic Medications.

Medication	Adverse Effect
Anti-inflammatory	
Antihistamines	Dry eye decreased accommodation, mydriasis
Corticosteroids	Ocular hypertension, open-angle glaucoma, cataracts, central serous chorioretinopathy **Routine screening is recommended to detect ocular hypertension and glaucoma in chronic users.**
Indomethacin	Corneal opacities, retinopathy
Anti-microbial	
Cidofivir	Uveitis
Ethambutol	Optic neuropathy **Routine screening is recommended to detect optic neuropathy.**
Fluoroquinolones	Iris depigmentation (moxifloxacin), uveitis (moxifloxacin), retinal detachment, optic neuropathy (ciprofloxacin)
Rifabutin	Uveitis
Sulfonamides	Transient myopia, acute angle closure, uveitis
Tetracyclines	Intracranial hypertension
Cardiovascular	
Alpha-agonists	Intraoperative floppy iris syndrome
Amiodarone	Corneal deposits, optic neuropathy
Digoxin	Visual disturbances (including yellow discoloration of vision, photopsias)
Niacin	Macular edema
Chemotherapeutics	
Bortezimib	Chalazia, dry eye
BRAF inhibitors	Dry eyes, conjunctivitis, uveitis
Busulfan	Cataracts
Carmustine	Ischemic retinopathy
EGFR-inhibitors	Blepharitis, dry eye, corneal epitheliopathy
Fluorouracil	Blepharitis, dry eye
Imatinib	Periorbital edema, macular edema, optic neuritis
Immune checkpoint inhibitors	Ophthalmoplegia (pembrolizumab), orbital inflammation, dry eye, uveitis, serous retinal detachments (Vogt-Koyanagi-Harada disease-like syndrome)
Interferons	Serous retinal detachments (Vogt-Koyanagi-Harada disease-like syndrome), ischemic retinopathy, optic neuropathy
MEK inhibitors	Dry eye, retinal vein occlusions, central serous retinopathy
Platinum analogs	Ischemic retinopathy, optic neuritis
Tamoxifen	Corneal deposits, cataracts, crystalline retinopathy, macular edema
Taxanes	Epiphora, macular edema, scintillating scotomas, optic neuropathy
Vincristine	Cranial nerve palsies, optic neuropathy
Immunomodulatory	
Chloroquine/Hydroxychloroquine	Maculopathy **Routine screening is recommended to detect maculopathy.**
Fingolimod	Macular edema **Routine screening is recommended to detect macular edema.**
Neurologic	
Amantadine	Corneal edema
Carbamazepine	Ocular motility dysfunction
Phenytoin	Ocular motility dysfunction
Topiramate	Acute angle closure glaucoma, transient myopia, uveitis, oculogyric crisis
Vigabatrin	Visual field loss **Routine screening is recommended to detect visual field loss.**
Psychiatric	
Phenothiazines	Eyelid and conjunctival pigmentation, corneal deposits, corneal epitheliopathy, corneal edema, cataracts, retinopathy
Selective serotonin reuptake inhibitors	Decreased accommodation, acute angle closure, cataracts
Tricyclic antidepressants	Mydriasis, decreased accommodation, acute angle closure
Other	
Allopurinol	Cataracts
Bisphosphonates	Episcleritis, scleritis, keratitis, uveitis
Defuroxamine	Retinopathy
Oral Contraceptives	Dry eye, uveitis, retinal vascular occlusion
Pentosan polysulfate sodium	Maculopathy **Routine screening is recommended to detect maculopathy**.
Phosophodiesterase-5 inhibitors	Blue discoloration of vision, non-arteritic ischemic optic neuropathy, retinal vascular occlusions
Thiazolidinediones (glitazones)	Macular edema
Vitamin A/Retinoids	Blepharoconjunctivitis, dry eye, chalazia, corneal opacities, intracranial hypertension

**Table 2:** Routine Screening for Ocular Toxicity in Select Systemic Medications.

Medication	Recommended Screening
Chloroquine/Hydroxychloroquine	Baseline testing prior to or within one year of initiation. Annual screening beginning after 5 years of treatment (sooner for high-risk patients) [9]. Testing: VA, DFE, macular OCT, and VF. FAF and mfERG may also be useful. Risk factors: >2.3 mg/kg/day (CQ) or >5.0 mg/kg/day (HCQ), renal disease, tamoxifen use, or macular disease.
Corticosteroids	While no formal screening guidelines exist, routine evaluation is recommended for IOP measurement and glaucoma screening in patients on chronic systemic corticosteroid treatment.
Ethambutol	Baseline examination (VA and color vision) at the time of initiation. Subsequent testing every 2 months while on treatment and regular self-assessments (VA and Amsler grid) have also been recommended [19]. Testing: VA, color vision, contrast sensitivity, VF, and OCT RNFL.
Fingolimod	Baseline testing prior to initiation is followed by repeat screening every 3–4 months and annually thereafter [36]. Testing: VA, DFE, and macular OCT.
Pentosan polysulfate sodium	Baseline testing prior to initiation is followed by annual monitoring as the cumulative dose approaches 500 g (~5 years at standard dosing) [13]. Testing: VA, DFE, macular OCT, FAF, NIR.
Vigabatrin	Baseline testing prior to initiation is followed by screening every 3 months during therapy and then 3 to 6 months after discontinuation [40,41]. Testing: VA and DFE. VF, ERG, or OCT RNFL depending on patient’s age.

Abbreviations: CQ = chloroquine; DFE = dilated fundus examination; ERG = electroretinography; FAF = fundus autofluorescence; HCQ = Hydroxychloroquine; IOP = intraocular pressure; mfERG = multifocal electroretinography; NIR = near-infrared; OCT = optical coherence tomography; RNFL= retinal nerve fiber layer; VA = visual acuity; VF = visual field.

**Table 3:** Incidences of Select Chemotherapeutic-Related Vision-Threatening Adverse Effects.

Medication	Incidence of Adverse Effect
Busulfan	Cataracts: 10–79% [42].
Carmustine	Ischemic retinopathy: 25% [43].
BRAF inhibitors	Uveitis: 4% [44].
Immune checkpoint inhibitors	Uveitis: 1% [45].
Interferons	Ischemic retinopathy: 20% [46].
MEK inhibitors	Retinal vein occlusion: 0.2% [47]. Central serous retinopathy 5–75% [48,49,50].

## Data Availability

Not applicable.

## References

[1] Alderaan K., Sekicki V., Magder L.S., Petri M. (2014). Risk factors for cataracts in systemic lupus erythematosus (SLE). Rheumatol. Int..

[2] Bensch G.W. (2016). Safety of intranasal corticosteroids. Ann. Allergy Asthma Immunol..

[3] Daniel B.S., Orchard D. (2015). Ocular side-effects of topical corticosteroids: What a dermatologist needs to know. Australas. J. Dermatol..

[4] Haeck I.M., Rouwen T.J., Mik L.T.-D., de Bruin-Weller M.S., Bruijnzeel-Koomen C.A. (2011). Topical corticosteroids in atopic dermatitis and the risk of glaucoma and cataracts. J. Am. Acad. Dermatol..

[5] Armaly M.F., Becker B. (1965). Intraocular pressure response to topical corticosteroids. Fed. Proc..

[6] Roberti G., Oddone F., Agnifili L., Katsanos A., Michelessi M., Mastropasqua L., Quaranta L., Riva I., Tanga L., Manni G. (2020). Steroid-induced glaucoma: Epidemiology, pathophysiology, and clinical management. Surv. Ophthalmol..

[7] Rosenbaum J.T., Costenbader K.H., Desmarais J., Ginzler E.M., Fett N., Goodman S.M., O’Dell J.R., Schmajuk G., Werth V.P., Melles R.B. (2021). American College of Rheumatology, American Academy of Dermatology, Rheumatologic Dermatology Society, and American Academy of Ophthalmology 2020 Joint Statement on Hydroxychloroquine Use With Respect to Retinal Toxicity. Arthritis Rheumatol..

[8] Melles R.B., Marmor M.F. (2014). The risk of toxic retinopathy in patients on long-term hydroxychloroquine therapy. JAMA Ophthalmol..

[9] Marmor M.F., Kellner U., Lai T.Y., Melles R.B., Mieler W.F. (2016). Recommendations on Screening for Chloroquine and Hydroxychloroquine Retinopathy (2016 Revision). Ophthalmology.

[10] Pearce W.A., Chen R., Jain N. (2018). Pigmentary Maculopathy Associated with Chronic Exposure to Pentosan Polysulfate Sodium. Ophthalmology.

[11] Hanif A.M., Armenti S.T., Taylor S.C., Shah R.A., Igelman A.D., Jayasundera K.T., Pennesi M.E., Khurana R.N., Foote J.E., O’Keefe G.A. (2019). Phenotypic spectrum of pentosan polysulfate sodium–associated maculopathy: A multicenter study. JAMA Ophthalmol..

[12] Vora R.A., Patel A.P., Melles R. (2020). Prevalence of Maculopathy Associated with Long-Term Pentosan Polysulfate Therapy. Ophthalmology.

[13] Wang D., Au A., Gunnemann F., Hilely A., Scharf J., Tran K., Sun M., Kim J.-H., Sarraf D. (2020). Pentosan-associated maculopathy: Prevalence, screening guidelines, and spectrum of findings based on prospective multimodal analysis. Can. J. Ophthalmol..

[14] Wang A.-G., Cheng H.-C. (2016). Amiodarone-Associated Optic Neuropathy: Clinical Review. Neuro-Ophthalmol..

[15] Passman R.S., Bennett C.L., Purpura J.M., Kapur R., Johnson L.N., Raisch D.W., West D.P., Edwards B.J., Belknap S.M., Liebling D.B. (2012). Amiodarone-associated Optic Neuropathy: A Critical Review. Am. J. Med..

[16] Wang M.Y., Sadun A.A. (2013). Drug-Related Mitochondrial Optic Neuropathies. J. Neuro-Ophthalmol..

[17] Chamberlain P.D., Sadaka A., Berry S., Lee A.G. (2017). Ethambutol optic neuropathy. Curr. Opin. Ophthalmol..

[18] Chen H.-Y., Lai S.-W., Muo C.-H., Chen P.-C., Wang I.-J. (2012). Ethambutol-induced optic neuropathy: A nationwide population-based study from Taiwan. Br. J. Ophthalmol..

[19] Saxena R., Singh D., Phuljhele S., Kalaiselvan V., Karna S., Gandhi R., Prakash A., Lodha R., Mohan A., Menon V. (2021). Ethambutol toxicity: Expert panel consensus for the primary prevention, diagnosis and management of ethambutol-induced optic neuropathy. Indian J. Ophthalmol..

[20] Lakshmi K., Ambika S., Gopal M., Noronha O. (2022). Visual outcomes of toxic optic neuropathy secondary to Ethambutol: A retrospective observational study from India, an endemic country. Indian J. Ophthalmol..

[21] Talbert E.K. (2010). Risk factors for ethambutol optic toxicity. Int. Ophthalmol..

[22] Wu A., Khawaja A.P., Pasquale L.R., Stein J.D. (2019). A review of systemic medications that may modulate the risk of glaucoma. Eye.

[23] Abtahi S.-H., Abtahi M.-A., Fazel F., Jenab K., Roomizadeh P., Etemadifar M., Akbari M. (2012). Topiramate and the vision: A systematic review. Clin. Ophthalmol..

[24] Fraunfelder F., Keates E.U. (2004). Topiramate-associated acute, bilateral, secondary angle-closure glaucoma. Ophthalmology.

[25] Fraser C.L., Biousse V., Newman N.J. (2012). Minocycline-Induced Fulminant Intracranial Hypertension. Arch. Neurol..

[26] Orme D.R., Vegunta S., Miller M.A., Warner J.E., Bair C., McFadden M., Crum A.V., Digre K.B., Katz B.J. (2020). A Comparison Between the Clinical Features of Pseudotumor Cerebri Secondary to Tetracyclines and Idiopathic Intracranial Hypertension. Am. J. Ophthalmol..

[27] Passi S.F., Butcher R., Orme D.R., Warner J.E., Stoddard G.J., Crum A.V., Gouripeddi R., Kirk B.H., Digre K.B., Katz B.J. (2022). Increased Incidence of Pseudotumor Cerebri Syndrome Among Users of Tetracycline Antibiotics. J. Neuro-Ophthalmol..

[28] Chisholm J.T., Abou-Jaoude M.M., Hessler A.B., Sudhakar P. (2017). Pseudotumor Cerebri Syndrome with Resolution After Discontinuing High Vitamin A Containing Dietary Supplement: Case Report and Review. Neuro-Ophthalmol..

[29] Lombaert A., Carton H. (1976). Benign intracranial hypertension due to A-hypervitaminosis in adults and adolescents. Eur. Neurol..

[30] Fraunfelder F., Corbett J.J. (2004). Isotretinoin-associated intracranial hypertension. Ophthalmology.

[31] Fraunfelder F.W. (2004). Evidence for a Probable Causal Relationship Between Tretinoin, Acitretin, and Etretinate and Intracranial Hypertension. J. Neuro-Ophthalmol..

[32] Cai S., Liu T.Y.A., Arevalo J.F. (2019). Evolution of Ellipsoid Zone Abnormalities on Optical Coherence Tomography Associated With Niacin Maculopathy. JAMA Ophthalmol..

[33] Domanico D., Verboschi F., Altimari S., Zompatori L., Vingolo E.M. (2015). Ocular Effects of Niacin: A Review of the Literature. Med. Hypothesis Discov. Innov. Ophthalmol..

[34] Mandal P., Gupta A., Fusi-Rubiano W., A Keane P., Yang Y. (2016). Fingolimod: Therapeutic mechanisms and ocular adverse effects. Eye.

[35] Zarbin M.A., Jampol L.M., Jager R.D., Reder A.T., Francis G., Collins W., Tang D., Zhang X. (2013). Ophthalmic Evaluations in Clinical Studies of Fingolimod (FTY720) in Multiple Sclerosis. Ophthalmology.

[36] Jain N., Bhatti M.T. (2012). Fingolimod-associated macular edema: Incidence, detection, and management. Neurology.

[37] Jonsson H., Lehto M., Vanhatalo S., Gaily E., Linnankivi T. (2021). Visual field defects after vigabatrin treatment during infancy: Retrospective population-based study. Dev. Med. Child Neurol..

[38] Foroozan R. (2018). Vigabatrin: Lessons Learned From the United States Experience. J. Neuro-Ophthalmol..

[39] Plant G.T., Sergott R.C. (2011). Understanding and interpreting vision safety issues with vigabatrin therapy. Acta Neurol. Scand..

[40] Sergott R.C., Wheless J.W., Smith M.C., Westall C.A., Kardon R.H., Arnold A., Foroozan R., Sagar S.M. (2010). Evidence-based Review of Recommendations for Visual Function Testing in Patients Treated with Vigabatrin. Neuro-Ophthalmol..

[41] Vigabatrin: The Problem of Monitoring for Peripheral Vision Loss in Children. https://www.aapos.org/HigherLogic/System/DownloadDocumentFile.ashx?DocumentFileKey=781a381d-6dae-ede4-bcf7-a3db9973bf15.

[42] Xu S., Tang X., Xu H., Bo F., Tang X. (2010). Total body irradiation plus cyclophosphamide versus busulphan with cyclophosphamide as conditioning regimen for patients with leukemia undergoing allogeneic stem cell transplantation: A meta-analysis. Leuk. Lymphoma.

[43] Johnson D., Cagnoni P., Schossau T., Stemmer S., Grayeb D., Baron A., Shpall E., Bearman S., McDermitt J., Jones R. (1999). Optic disc and retinal microvasculopathy after high-dose chemotherapy and autologous hematopoietic progenitor cell support. Bone Marrow Transplant..

[44] Choe C.H., McArthur G.A., Caro I., Kempen J.H., Amaravadi R.K. (2014). Ocular Toxicity in BRAF Mutant Cutaneous Melanoma Patients Treated With Vemurafenib. Am. J. Ophthalmol..

[45] Ribas A., Hamid O., Daud A., Hodi F.S., Wolchok J.D., Kefford R., Joshua A.M., Patnaik A., Hwu W.-J., Weber J.S. (2016). Association of Pembrolizumab With Tumor Response and Survival Among Patients With Advanced Melanoma. JAMA.

[46] Lim J.W., Shin M.-C. (2009). Pegylated-Interferon-Associated Retinopathy in Chronic Hepatitis Patients. Ophthalmologica.

[47] Infante J.R., Fecher L.A., Falchook G.S., Nallapareddy S., Gordon M.S., Becerra C., DeMarini D.J., Cox D.S., Xu Y., Morris S.R. (2012). Safety, pharmacokinetic, pharmacodynamic, and efficacy data for the oral MEK inhibitor trametinib: A phase 1 dose-escalation trial. Lancet Oncol..

[48] Urner-Bloch U., Urner M., Stieger P., Galliker N., Winterton N., Zubel A., Parseval L.M.-D., Dummer R., Goldinger S.M. (2014). Transient MEK inhibitor-associated retinopathy in metastatic melanoma. Ann. Oncol..

[49] Ruud van der Noll R., Leijen S., Neuteboom G.H., Beijnen J.H., Schellens J.H. (2013). Effect of inhibition of the FGFR–MAPK signaling pathway on the development of ocular toxicities. Cancer Treat. Rev..

[50] Van Dijk E.H., Van Herpen C.M., Marinkovic M., Haanen J.B., Amundson D., Luyten G.P., Jager M.J., Kapiteijn E.H., Keunen J.E., Adamus G. (2015). Serous retinopathy associated with mitogen-activated protein kinase kinase inhibition (binimetinib) for metastatic cutaneous and uveal melanoma. Ophthalmology.

